# Monitoring of sausage products sold in Sichuan Province, China: a first comprehensive report on meat species’ authenticity determination

**DOI:** 10.1038/s41598-019-55612-x

**Published:** 2019-12-13

**Authors:** Qiuchi Song, Yiwu Chen, Liming Zhao, Hongsheng Ouyang, Jun Song

**Affiliations:** 1 Analytical and Testing Center, Sichuan Academy of Agricultural Sciences, Chengdu, 610066 P.R. China; 2 College of Agronomy, Sichuan Agricultural University Chengdu, Chengdu, 611130 P.R. China; 3 College of Animal Science, Jilin University, Changchun, 130062 P.R. China; 4 Personnel and Labor Department, Sichuan Academy of Agricultural Sciences, Chengdu, 610066 P.R. China

**Keywords:** Biotechnology, Risk factors

## Abstract

Presently, there is growing concern worldwide regarding the adulteration of meat products. However, no reports on determining meat authenticity have been reported in China. To verify labelling compliance and evaluate the existence of fraudulent practices, 250 sausage samples were purchased from local markets in Sichuan Province and analysed for the presence of chicken, pork, beef, duck and genetically modified soybean DNA using real-time and end-point PCR methods, providing a Chinese case study on the problem of world food safety. In total, 74.4% (186) of the samples were properly labelled, while the other 25.6% (64) were potentially adulterated samples, which involved three illicit practices: product removal, addition and substitution. The most common mislabelling was the illegal addition of, or contamination with, duck. Therefore, meat authenticity monitoring should be routinely conducted. Additionally, the strict implementation of the nation’s food safety laws, along with regular surveillance, should be compulsory to alleviate and deter meat adulteration.

## Introduction

The meats of swine, cattle, sheep and poultry are currently consumed by people in many countries, including China, as main sources of animal protein^1^. Currently, China is the largest producer of meat in the world^2,3^, even though the quantity of meat consumed per capita per year in China is not the greatest. With the increase in consumer incomes in China, the structure and quantity of meat consumption have changed greatly in recent years. In 2014, the consumed quantities of swine, both cattle and sheep, and poultry meat per capita in China were 20, 1.6 and 8 kg^4^, respectively. When compared with the values in 2000 in China, the consumption per capita of swine and poultry meat increased by 37.65% and 113%, respectively, while that of cattle and sheep fell 17.1%^4^. In general, meat consumption per capita has grown in China over the past 30 years. Significantly, the quantity of porcine meat consumed is decreasing, while the consumption of cattle, sheep and poultry is increasing steadily. Currently, the quantity of meat consumed in China is at a moderate level compared with other countries. The characteristics of meat consumption in China are similar to those of Hong Kong, Taiwan and Japan, which are different from those of the USA^5^.

Sichuan Province in southwest China is located in the upper reaches of the Yangtze River. With Chengdu as the capital city, Sichuan covers an area of over 486,000 km^2^ and ranks fifth in China in terms of area, having jurisdiction over 21 cities (states) and 183 counties (county-level cities and districts). Sichuan has a population of ~82.26 million, of which 100,000 are members of the Hui Muslim minority. The members of the Muslim Hui community are distributed in all the counties of Sichuan Province, but mainly in Chengdu (CD), Aba (AB), Mianyang (MY) and Xichang (XC) cities^6^. The Hui Muslim minority follows strict dietary laws enshrined in the holy Quran. Under their religious rules, Hui Muslim people are forbidden to eat pork, and therefore, its consumption must be avoided. In 2015, the meat consumption per capita in Sichuan was the highest in China, with a value of 39.3 kg^5^.

With the exposure of the horse meat scandal in Europe, modern consumers are increasingly health conscious and are demanding more comprehensive information on the origin, composition and safety of the foods they consume^7–9^, and the authenticity of halal foods has also attracted the attention of the Hui Muslim people. Now, the authenticity determination of meat products in the Middle East and other Islamic countries, especially in East Asia, is mandatory^10^ owing to fraudulent practices, such as adulteration, substitution and mislabelling, in the production of meat-containing products. However, the Department of Supervision and Administration for Food Safety in China currently focuses on the “safety” aspects of meat products, including the presence of veterinary drug residues, pathogenic microorganisms, heavy metals and chemical additives exceeding the regulatory limits, while the authenticity of meat products is not emphasised.

Authenticity is a paramount important criterion for food safety and quality. However, the identities of the ingredients in processed or composite mixtures are not always readily apparent^11^. Recent food scares, frauds and malpractices carried out by some food producers have increased public interest in the composition of food products owing to religious reasons and food allergies^12^. Consumers have the right to know what is in the food they are eating, and producers have a duty to inform and not knowingly mislead consumers regarding the sources or contents of their food products^13^. Food fraud is defined as “the deliberate and intentional substitution, addition, tampering, or misrepresentation of food, food ingredients, or food packaging; or false or misleading statements made about a product, for economic gain”, and it greatly affects food safety and public health^14,15^. Three common categories of food fraud are replacement, addition and removal. Among these, substitution is the most common, followed by addition^14^. Among foods, processed meat products, like ham sausages, are particularly vulnerable to adulteration because minced meat production removes the morphological characteristics of muscle, making it difficult to identify the origin^16^. Therefore, ham sausage can be illegally adulterated, substituted and added to in many ways during the production and distribution chains. Chinese ham sausage is a complex mixture, consisting mainly of meat and starch, as well as low concentrations of water, vegetable oil, salt, monosodium glutamate and other food additives. Additionally, chicken, duck and goose meats are cheap and widely available in Chinese markets. Consequently, to enhance profits, these cheaper meats are commonly adulterated into ham sausage^16^. Pork, which cannot be consumed under Muslim and Jewish dietary restrictions, is the most commonly substituted meat in South Africa^17^. At present no studies on meat substitution in the Sichuan market of China have been reported. Therefore, there is a need to investigate meat species to verify that the products are in compliance with the label statements and regulations in force, and to support fair trade and protect consumer rights^18^.

By 2016, China had approved 60 genetically modified (GM) crop events for food and feed use^19^. Of the GM soybean events, GTS40-3-2, MON89788 and A2704-12 are the most imported transgenic soybean lines in China^20^. Soybean powder is commonly added to ham sausage as a raw material because its addition makes the structure of the sausage tight and elastic, as well as rendering the cut surface smooth. Thus, it improves the overall quality of sausage products^21^. According to the administrative regulations on the safety of GM organisms in China, the labelling of GM products or food is mandatory. Several studies worldwide have found different degrees of species adulteration in meat products^17,22–27^ but no similar studies in China have been published. Therefore, there is currently a lack of information on adulterated meat products. Owing to the stability of DNA, most analytical methods used to date for meat authentication have relied on the detection of species-specific DNA^28^, and various PCR-based studies have been suggested by researchers^29^. In the past decade, PCR methods, such as end-point and real-time PCR, have been widely used to detect adulterated meat products^30–34^. To verify labelling compliance and evaluate the existence of fraudulent practices, 250 ham sausage samples were purchased from local markets in 21 cities of Sichuan Province, and they were, for the first time, analysed for the presence of chicken, swine, cattle, duck and GM soybean DNA using real-time and end-point PCR methods. The aims of this study were to detect the presence of species substitutions, undeclared species, species cross-contamination and GM ingredients in ham sausages, to investigate the incidence of mislabelling in ham sausages sold in Sichuan, China and to assess the impacts of such practices on consumer confidence and fair trade.

## Materials and Methods

### Sample materials

**Figure 1:**
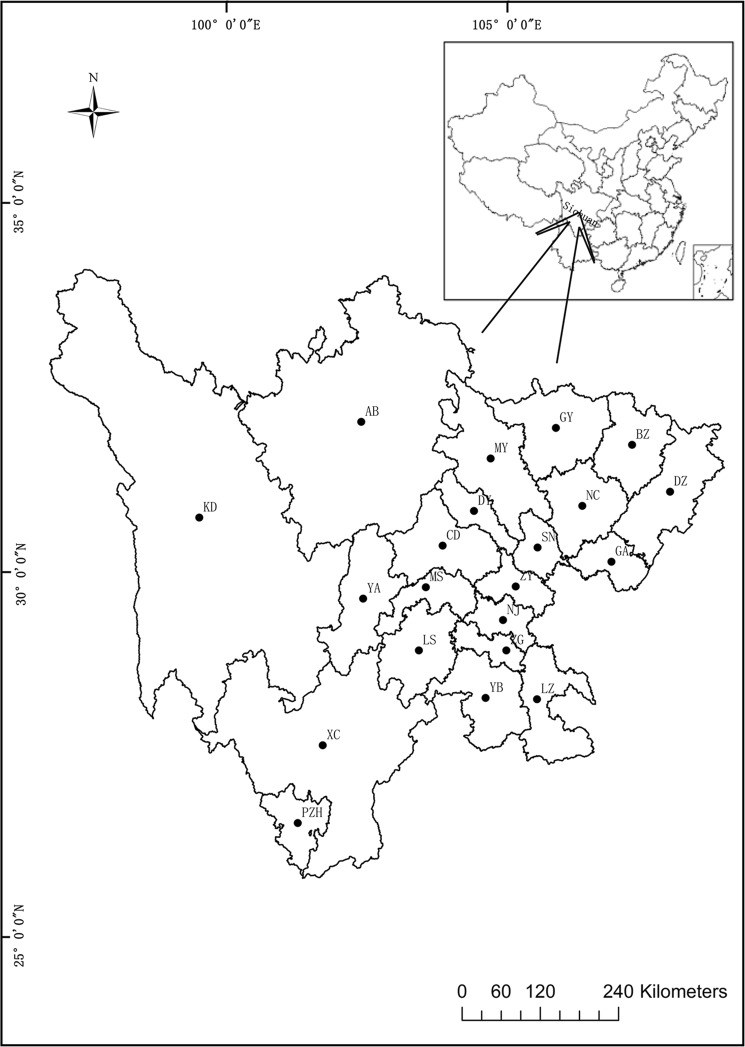
Geographical distribution of markets from which the sampled sausage products were purchased for this study. Locality codes correspond to the city names in Table 1.

A total of 250 sausage products, representing a variety of meat origins (pork, chicken, duck, beef or mixtures) were purchased, between July and August 2016, from 77 local markets and 89 restaurants across 21 counties in Sichuan Province (Fig. 1). These samples represented six different types of meat or meat mixtures, including products labelled as containing chicken (n = 16; Type I) and mixtures of chicken and duck (n = 47; Type II), chicken and pork (n = 156; Type III), chicken, duck and pork (n = 29; Type IV), chicken, pork and beef (n = 1; Type V), and chicken, duck and beef (n = 1; Type VI). Sample details are shown in Table 1. Manufacturer names are not disclosed. All the samples were frozen and maintained at 4 °C in our laboratory. The authentic meat samples (reference samples) of swine, chicken, duck and cattle used as positive controls were purchased from the Chinese Academy of Inspection and Quarantine (Beijing, China). The reference materials for GM soybean GTS40-3-2 were purchased from the Institute for Reference Materials and Measurements in Geel, Belgium, and the reference DNAs for GM soybean A2704-12 and MON89788 were purchased from the American Oil Chemists Society (Champaign-Urbana, IL, USA).

**Table 1:** Sampling localities, locational abbreviations, and the numbers and identities of samples purchased from local markets in Sichuan, China.

Locality	Code for the locations	Designing of sampling strategy		Sample identity	Sample size (n)	No. of samples mislabelling the meat ingredients	No. of samples testing positive for 3 GM soybeans		
		No. of supermarkets or retails	No. of restaurants				GTS40-3-2	MON89788	A2704-12
Chengdu	CD	8	6	CD1- CD16	16	3	0	0	0
Panzhihua	PZH	4	5	PZH1-PZH12	12	9	0	0	0
Xichang	XC	5	3	XC1-XC11	10	5	0	0	0
Dazhou	DZ	8	4	DZ1-DZ12	12	5	0	0	0
Guangan	GA	4	6	GA1-GA12	12	4	0	0	0
Suining	SN	3	6	SN1-SN12	12	1	0	0	0
Kangding	KD	2	3	KD1-KD10	10	6	0	0	0
Yibing	YB	2	6	YB1-YB12	12	1	0	0	0
Luzhou	LZ	4	6	LZ1-LZ12	12	0	0	0	0
Meishan	MS	2	5	MS1-MS12	12	1	0	0	0
Leshan	LS	3	6	LS1-LS12	12	3	0	0	0
Nanchong	NC	4	6	NC1-NC12	12	1	0	0	0
Bazhong	BZ	4	3	BZ1-BZ12	12	0	0	0	0
Guangyuan	GY	4	1	GY1-GY12	12	9	0	0	0
Mianyang	MY	2	3	MY1-MY12	12	2	0	0	0
Deyang	DY	2	2	DY1-DY12	12	2	0	0	0
Yaan	YA	2	6	YA1-YA12	12	4	0	0	0
Zigong	ZG	5	3	ZG1-ZG12	12	0	0	0	0
Neijiang	NJ	3	5	NJ1-NJ12	12	6	0	0	0
Ziyang	ZY	2	4	ZY1-ZY12	12	0	0	0	0
Aba	AB	4	0	AB1-AB10	10	2	0	0	0
Total		77	89		250	64	0	0	0

### DNA extraction

Genomic DNAs were isolated and purified from the reference materials and test samples, and sterilised ultrapure water was used as a negative control (blank) for DNA extraction using a genomic DNA purification kit (Tiangen Biotech Co., Ltd., Beijing, China) following the manufacturer’s instructions. The DNA concentrations were measured using a NanoDrop ND-1000 UV Spectrophotometer (Thermo Fisher Scientific, Waltham City, MA, USA).

### Primers and PCR conditions

Species-specific DNA segments for duck, pork and beef, and the 18S rRNA of eukaryotes (control for species DNA) were amplified using primer and probe sequences as described in the Inspection and Quarantine Industry Standard of the People’s Republic of China^35,36^ and the National Standard of the People’s Republic of China^37^ (Table 2). DNA species-specific fragments of duck, pork and beef, as well as the 18S rRNA of eukaryotes, in samples and reference materials were amplified using real-time PCR on an ABI 7500 fluorometric thermal cycler (Applied Biosystems, Foster City, CA, USA), with simultaneous amplification of the blank from the DNA extraction and non-template PCR control. All the samples were tested for the presence of duck-, pork- and beef-specific DNA fragments. The real-time PCR assays were carried out in a final volume of 25 µL containing 1× THUNDERBIRD™ Probe qPCR Mix (Toyobo Co., Ltd., Osaka, Japan), 1× ROX reference dye, 500 nM each primer, 250 nM probe and approximately 50–100 ng genomic DNA per sample or reference material (control) as the template^35–37^. The thermocycling settings for beef were as follows: initial incubation at 95 °C for 10 min and then 45 cycles of 95 °C for 15 s and 60 °C (annealing and extension, respectively) for 1 min. Fluorescence measurements were taken after annealing and extension. For the thermal amplification programs of pork and duck, the annealing temperature was set to 58 °C for 15 s, and all other program parameters were the same as those for beef.

**Table 2:** Primers and probes used in the PCR analyses.

Primer (probe) name	Sequence (5′-3′)	Amplicon size(bp)	Type of PCR	objective	References
Lectin-F	GCCCTCTACTCCACCCCCATCC	118	End point PCR	Endogenous gene of soybean analysis	Liu *et al*., 2012
Lectin-R	GCCCATCTGCAAGCCTTTTTGTG				
GTS-40-3-2-F	TTCAAACCCTTCAATTTAACCGAT	370	End point PCR	GTS-40-3-2 event-specific analysis	Liu *et al*., 2012
GTS-40-3-2-P	AAGGATAGTGGGATTGTGCGTC				
MON89788-F	CTGCTCCACTCTTCCTTT	223	End point PCR	MON89788 event-specific analysis	Zhang *et al*., 2010
MON89788-R	AGACTCTGTACCCTGACCT				
A2704-12-F	TGAGGGGGTCAAAGACCAAG	239	End point PCR	A2704-12 event-specific analysis	Yang *et al*., 2010
A2704-12-R	CCAGTCTTTACGGCGAGT				
Chicken-F	CTATAATCGATAATCCACGATTCA	131	End point PCR	Chicken species-specific analysis	Zong, *et al*., 2011
Chicken-R	CTTGACCTGTCTTATTAGCGAGG				
Porcine-F	ATCTACATGATTCATTACAATTAC	68	Real time PCR	Porcine species-specific analysis	Gao, *et al*., 2013
Porcine-R	CTATGTTTTTGAGTTTTGAGTTCA				
Porcine-P	FAM-ATCTCAAACTACTCATACCCA-TAMARA				
Duck-F	AAGCCTTCCTCTAGCTCAGC	65	Real time PCR	Duck species-specific analysis	Yu, *et al*., 2010
Duck-R	AGAAAATGCTTTAGTTAAGTC				
Duck-P	FAM-CTCAGCCGCTTAAACAACGC-TAMARA				
Beef-F	CCGATGGATGTGTTCAGAGCT	70	Real time PCR	Beef species-specific analysis	Chen, *et al*., 2010
Beef-R	GCCAAATGTCTGGGTGTAGATACC				
Beef-P	FAM-TCGGCTTTAGGGCTTCCGAATGTGAA-TAMARA				
18SrRNA-F	TCTGCCCTATCAACTTTCGATGGTA	91	Real time PCR	18S rRNA of eukaryote analysis	Chen, *et al*., 2010
18SrRNA-P	FAM-ATCTCAAACTACTCATACCCA-TAMARA				
18SrRNA-R	AATTTGCGCGCCTGCTGCCTTCCTT				

The sequences of primer pairs for the amplification of chicken, the three GM soybean events (GTS40-3-2, A2704-12 and MON89788) and the endogenous Lectin gene (soybean taxon-specific gene used as a control for soybean DNA) were from data in the Inspection and Quarantine Industry Standard of the People’s Republic of China^38^ and the Agricultural Industry Standard of the People’s Republic of China^39–41^. All of the primers and probes (Table 2) were synthesised by Sangon Biotechnology Co, Ltd. (Shanghai, China). All of the end-point PCR (conventional PCR) reactions were carried out in an ABI Veriti 96 thermal cycler (Applied Biosystems) with a 25-µL reaction volume, containing approximately 50 ng of genomic DNA, 1× QuickTaq^®^ HS DyeMix (Toyobo Co., Ltd.) and 400 nM of each primer^38–41^. The amplification of the blank for DNA extraction and non-template PCR control were performed simultaneously with the same primers to determine whether contamination occurred during DNA extraction and PCR set up. The thermocycling program of the conventional PCR assays for the Lectin gene and two GM soybean lines, GTS-40-3-2 and A2704-12, were as follows: initial denaturation for 5 min at 95 °C; 35 cycles of amplification for 30 s at 94 °C, 30 s at 58 °C and 30 s at 72 °C; and a final extension for 7 min at 72 °C. The annealing temperatures of amplification for the MON89788 line and chicken species-specific DNA were 56 °C and 63 °C, respectively. Except for the annealing temperatures, the other program parameters were the same as those of the GM soybean lines GTS-40-3-2 and A2704-12. As indicated by the standards^35–41^, the specificity of the methods described was highly specific, and the limit of detection was 0.1%. The specificity (high specificity) and sensitivity (five copies) levels of the conventional and real-time PCR methods used to analyse the sausage samples were not evaluated in this study because they have been previously confirmed as the national standards for GM organism detection in China. PCR products were analysed by 2.5% agarose gel electrophoresis containing ethidium bromide in 1× Tris-acetate EDTA buffer for 20 min at 110 V.

## Results

There were no targeted DNA bands or amplification signals in the PCR of the blank controls, and the expected DNA bands or amplification signals were obtained in the PCR of the positive controls. In total, 250 sausage samples collected in 166 local markets or restaurants in 21 cities in Sichuan Province, China were first identified using PCR methods. DNA fragments of the 18S rRNA and/or Lectin gene were amplified successfully from templates isolated from all the samples, indicating the efficient extraction of genomic DNA from eukaryotes (meat species) and soybean (plant addition to sausage), which can be used in the subsequent analyses. Among the samples, the mislabelling of 64 products (25.6%) was found. Mislabelled samples collected from 17 cities, except for sausage samples purchased from LZ, BZ, ZY and ZG, were determined (Table 1). The greatest frequency (75.0%) of mislabelling was observed in PZH, and the lowest frequencies (8.3%) were detected in SN, MS and NC. Although most of the sausage products sampled were from the capital city of Sichuan Province (CD), the frequency of mislabelled samples was less than 19%. Surprisingly, the three highly imported GM soybean lines, GTS40-3-2, MON89788 and A2740-12, were not detected in any sample labelled as containing soybean.

Among the 64 mislabelled samples, one sample labelled as Type III (containing chicken and pork) tested positive for chicken and duck (Type II; Table 3), representing a typical meat species substitution. Three samples labelled as Type I (containing chicken, duck and pork), tested negative for duck (Type III; Table 3). Additionally, a high incidence of illegal additions or contamination of undeclared meat species was observed in 60 samples. Duck meat was the most common undeclared animal species found in 58 samples, while unclaimed pork was detected in the 2 samples labelled as Types II and VI. The greatest incidences (75%) of illegal additions of, or contamination with, undeclared duck species were found in PZH and GY, followed by 60% in KD and 41.7% in DZ and NJ.

**Table 3:** Fraudulent substitution, removal and addition of meat ingredients in sausage samples from Sichuan, China.

Code for sampling locations	No. of mislabelled samples	No. of substituted samples	No. of samples illegally added or cross-contaminated with other animal species				No. of other samples with removal
			Porcine	Chicken	Duck	Beef	
CD	3	0	0	0	3	0	0
PZH	9	0	0	0	9	0	0
XC	5	0	0	0	3	0	2
DZ	5	0	0	0	5	0	0
GA	4	0	0	0	4	0	0
SN	1	0	0	0	1	0	0
KD	6	0	0	0	6	0	0
YB	1	0	0	0	1	0	0
LZ	0	0	0	0	0	0	0
MS	1	0	0	0	0	0	1
LS	3	0	0	0	3	0	0
NC	1	0	1	0	0	0	0
BZ	0	0	0	0	0	0	0
GY	9	0	0	0	9	0	0
MY	2	0	0	0	2	0	0
DY	2	0	0	0	2	0	0
YA	4	0	0	0	4	0	0
ZG	0	0	0	0	0	0	0
NJ	6	1	0	0	5	0	0
ZY	0	0	0	0	0	0	0
AB	2	0	1	0	1	0	0
total	64	1	2	0	58	0	3

Table 3 is grouped on the basis of sampling location.

Illegal additions or cross-contamination of undeclared duck meat were detected in 25% of Type I samples, with Ct values that ranged between 22.87 and 35.18. In addition, unclaimed pork was detected in Type II samples, with the lowest prevalence rate (2.1%). Additionally, duck meat was substituted for pork in one sample labelled as Type III. However, the presence of undeclared duck DNA was prevalent (34.6%) in the Type III sample group, with the Ct values for duck DNA being in the range of 20.75–35.47. Of all the Ct values for unclaimed duck, there were 31 Ct values of less than 27, and 25 Ct values that varied between 30 and 35. On the contrary, declared duck was not detected in 10.3% of Type IV samples. The Type V sample was correctly labelled. As in Type II, the illegal addition or contamination of undeclared pork meat existed in Type VI, with a Ct of 25.74 (Table 4).

**Table 4:** Sample groups and mislabelling, the fraudulent substitution, removal and addition of meat ingredients in sausage samples from Sichuan, China.

Group Type	Products labelled as different meat	No. of samples	No. of mislabelled samples	No. of substituted samples	No. of samples illegally added or cross-contaminated with other species				No. of other samples with removal	Ct value for mislabelled samples
					Porcine	Chicken	Duck	Beef		
I	products labelled as chicken	16	4	0	0	0	4	0	0	22.87–35.18
II	products labelled as mixture of chicken and duck	47	1	0	1	0	0	0	0	26.79
III	products labelled as mixture of chicken and porcine	156	55	1	0	0	54	0	0	20.75–35.47
IV	products labelled as mixture of chicken, duck and porcine	29	3	0	0	0	0	0	3	undetermined
V	products labelled as mixture of chicken, porcine and beef	1	0	0	0	0	0	0	0	—
VI	products labelled as mixture of chicken, duck and beef	1	1	0	1	0	0	0	0	25.74

Table 4 is grouped by product category to emphasise the number of mislabelling and the fraudulent substitution, removal and addition events of meat ingredients in sausage samples.

## Discussion

Here, for the first time, the meat authenticity of ham sausages produced in China was investigated to assess the extent of mislabelling in sausage products using two types of detection methods based on PCR. In total, 74.4% of the samples were properly labelled, with the contents corresponding to the product lists. However, 25.6% of the potentially adulterated samples were subject to one of three illicit practices, removal (declared species not detected in the product), addition and substitution with another species. The percentage of mislabelling (25.6%) in this work was relatively small compared with those discovered in American (38.5%)^42^ and Malaysian (78.3%)^43^ markets. However, the percentage of mislabelling (25.6%) was greater than that reported in Canadian markets (20%)^26^. The most common mislabelling in this study was contamination with, or the illegal addition of, duck.

Altogether, 58 samples were found to contain undeclared duck as determined by real-time PCR, with Ct values ranging from 20.75 to 35.47. However, it was difficult to determine whether duck meat was intentionally added to the 58 samples or these samples were illegally contaminated with duck meat because Ct values are affected by many factors (i.e., inhibitory ingredients in the samples and the level of DNA degradation). Similarly, two samples containing unstated pork had low Ct values of 25.74 and 26.79, which indicated illegal additions or cross-contamination. In the sausage production chain, cross-contamination can arise when improperly cleaned (or uncleaned) equipment is used to process meat from more than one species^44^. Perhaps, a quantitative PCR analysis will aid in determining whether undeclared ingredients are additives or contaminants. Compared with the illegal additives in sausage products reported in previous studies^17,22–24^, in China, the prevalent unlabelled additive to sausage was duck, while in other countries chicken was the major unlabelled additive. Generally, the average price of duck is low in China, while beef is the highest, followed by pork and chicken. Therefore, duck is an economical additive for manufacturers to decrease the production cost of sausages. The different prices of meats worldwide explained why the illegal meat species used as additives differ between the sausages produced in China and those in other countries. They also explained the substitution of duck for pork in the test samples. However, the more expensive pork was added to two samples labelled as Types II and VI, and they had pork-associated Ct values of less than 30. Accordingly, the presence of pork did not seem to be the result of cross-contamination events. Similarly, it is hard to understand why the declared duck was not detected in three samples marketed as Type IV. It may be that the two aforementioned cases were mislabelled as human errors.

Another main reason for performing a survey on the meat authentication of sausage is because of religious concerns. Of the 250 sausage samples, two were clearly listed as meeting Muslim dietary laws (Halal) on their labels. One tested positive for undeclared duck, while the other was correctly labelled. Fortunately, the pork prohibited by Halal was not detected. China is a multiracial country, with a Muslim minority population of more than 10 million. It is estimated that more than 100,000 Hui people live in Sichuan Province, China. The mislabelling of sausage products may lead to the violation of personal or religious beliefs. A previous study reported that pork is the most common meat substitute, and its consumption breaches Muslim dietary restrictions^17^. Unfortunately, pork was found in sausages and ground-meat products in previous investigations^10,17^. Although pork was not found in the Halal sausages tested in this study, the detection of meat origins for Halal products should not be neglected in the future.

China has imported large quantities of GM soybeans since 2000. However, the three most imported GM soybean lines (GTS40-3-2, MON89788 and A2704-12) were not detected in any of the sausage samples, as in highly processed foods in previous reports^45,46^. It is challenging to extract and amplify total DNA from heavily processed products (especially fermentation products) because of the degradation of genomic DNA. Thus, the exogenous DNA of GM soybean in the samples tested may have degraded during fermentation; therefore, the targeted DNA segments (flanking DNA fragments) were not amplified^46^.

The observations of illegal additions, removals and substitutions in sausages in this study were similar to those of previous research^17,22–24^. Because of the high market value of meat compared with most plant-derived products, meat products are often targets for adulteration^17^. The sausage production technique often leads to changes in the appearance, colour, texture and flavour of the meat; therefore, additions, removals and substitutions are easily masked^47^. Thus, it is very difficult to visually detect these illicit events during meat processing. Consequently, adulterations are commonly found in sausage and ground meat products^17,22–24^.

The accurate identification of ground-meat products, including sausages, in markets is a growing concern because of the high incidence of adulteration worldwide. This study revealed that the mislabelling of sausages owing to illegal additions, substitutions and removals is a reality in China, and that local consumers are undoubtedly encountering undeclared animal species in sausage products. Our results provide a baseline for the preliminary assessment of meat species’ mislabelling in sausage products in China. Owing to recent food fraud scandals, including the intentional contaminating of infant milk formula with melamine^48–50^, food safety in China currently focuses on identifying the presence of poisonous chemical substances in foods. The food control authorities in China are not routinely monitoring sausage and ground-meat products for mislabelling. To date, the authentication of meat species in meat products has not been conducted across China. Although the mislabelling resulting from additions, substitutions of undeclared meat species and the removal of declared meat species may not impact food safety and public health, consumers can be deceived regarding the products they are purchasing and consuming^51^. Presently, according to our research, the fraudulent mislabelling of the additional of duck to sausages is becoming an important problem in the meat industry. The meat industry in China will face a crisis in consumer confidence if the adulteration of sausages becomes generalised. Identifying the species’ origins in sausages and meat products is important for preventing adulteration and for protecting consumers’ health and religious convictions^23^.

In China, the adulteration and misbranding of meat products are prohibited by the related food safety laws. Despite the implementation of more stringent food labelling regulations locally and globally, the adulteration or misrepresentation of food products for illicit financial gain continues to be a common societal problem^52,53^. Accordingly, public safety measures and testing procedures should be implemented by regulatory agencies based on the declared products’ contents. This is an ongoing issue that urgently requires monitoring. This survey highlights the importance of increasing national concerns and government efforts in food traceability. The strict implementation of the food safety laws of the People’s Republic of China, along with regular surveillance and monitoring programs, are required to alleviate and deter mislabelling issues. Additionally, regular audits of processed meat plants by the regulatory agencies should be implemented to ensure that manufacturing operations comply with Chinese food regulations. While this investigation suggests the occurrence of the addition of undeclared meat species in sausage products, further studies are needed to detect the extent of mislabelling and to identify points in the production chain where mislabelling occurs. Future areas of work also include the expansion of the tested meat species, the ground meat products and the sampling sites across China.

## Conclusions

Although several regulations and laws related to food and agricultural products are enforced in China, there is still a lack of information on the authentication of meat species. This study found a relatively low incidence (25.6%) of mislabelling owing to the adulteration of sausage products on the commercial market compared with previous reports^17,22–24^. Nevertheless, further studies are needed to determine the extent of adulteration across the whole meat industry in China, which would provide the government with more comprehensive data to be used in decision-making related to controlling the quality and safety of meat products.

## Data Availability

The datasets generated or analysed during the current study have been provided in this manuscript.
